# A systematic review and meta-analysis of the efficacy and safety of iguratimod in the treatment of inflammatory arthritis and degenerative arthritis

**DOI:** 10.3389/fphar.2024.1440584

**Published:** 2024-10-10

**Authors:** Zhiyong Long, Liuting Zeng, Kailin Yang, Junpeng Chen, Yanfang Luo, Charles C. Dai, Qi He, Ying Deng, Anqi Ge, Xiaofei Zhu, Wensa Hao, Lingyun Sun

**Affiliations:** ^1^ Department of Physical Medicine and Rehabilitation, The Affiliated Panyu Central Hospital, Guangzhou Medical University, Guangzhou, China; ^2^ Department of Rheumatology and Immunology, Nanjing Drum Tower Hospital, Chinese Academy of Medical Sciences and Peking Union Medical College, Graduate School of Peking Union Medical College, Nanjing, China; ^3^ Key Laboratory of Hunan Province for Integrated Traditional Chinese and Western Medicine on Prevention and Treatment of Cardio-Cerebral Diseases, School of Integrated Chinese and Western Medicine, Hunan University of Chinese Medicine, Changsha, China; ^4^ Psychosomatic Laboratory, Department of Psychiatry, Daqing Hospital of Traditional Chinese Medicine, Daqing, China; ^5^ Department of Physiology, School of Medicine, University of Louisville, Louisville, KY, United States; ^6^ Tong Jiecheng Studio, Hunan University of Science and Technology, Xiangtan, China; ^7^ The Central Hospital of Shaoyang, Shaoyang, China; ^8^ Department of Oral and Maxillofacial Surgery, School of Dentistry, University of Maryland, Baltimore, MD, United States; ^9^ Fischell Department of Bioengineering, A.James Clark School of Engineering, University of Maryland, College Park, MD, United States; ^10^ People’s Hospital of Ningxiang City, Ningxiang, China; ^11^ Fudan University, Shanghai, China; ^12^ Institute of Materia Medica, Chinese Academy of Medical Sciences and Peking Union Medical College, Beijing, China

**Keywords:** inflammatory arthritis, iguratimod, systematic review, meta-analysis, degenerative arthritis

## Abstract

**Objective:**

To assess the efficacy and safety of iguratimod (IGU) in the treatment of inflammatory arthritis and degenerative arthritis.

**Methods:**

Initially, randomized controlled trials (RCTs) on using IGU in treating inflammatory arthritis and degenerative arthritis were systematically gathered from various databases up to February 2024. Subsequently, two researchers independently screened the literature, extracted data, assessed the risk of bias in included studies, and conducted a meta-analysis using RevMan 5.4 software.

**Results:**

Fifty-four RCTs involving three inflammatory arthritis were included, including ankylosing spondylitis (AS), osteoarthritis (OA), and rheumatoid arthritis (RA). For AS, the meta-analysis results showed that IGU may decrease BASDAI (SMD −1.68 [−2.32, −1.03], *P* < 0.00001) and BASFI (WMD −1.29 [−1.47, −1.11], *P* < 0.00001); IGU may also decrease inflammatory factor [ESR: (WMD −10.33 [−14.96, −5.70], *P* < 0.0001); CRP: (WMD −10.11 [−14.55, −5.66], *P* < 0.00001); TNF-α: (WMD −6.22 [−7.97, −4.47], *P* < 0.00001)]. For OA, the meta-analysis results showed that IGU may decrease VAS (WMD −2.20 [−2.38, −2.01], *P* < 0.00001) and WOMAC (WMD −7.27 [−12.31, −2.24], *P* = 0.005); IGU may also decrease IL-6 (WMD −8.72 [−10.00, −7.45], *P* < 0.00001). For RA, the meta-analysis results showed that IGU may improve RA remission rate [ACR20: (RR 1.18 [1.02, 1.35], *P* = 0.02); ACR50: (RR 1.32 [1.05, 1.64], *P* = 0.02); ACR70: (RR 1.44 [1.02, 2.04], *P* = 0.04)] and decrease DAS28 (WMD −0.92 [−1.20, −0.63], *P* < 0.00001); IGU may also decrease inflammatory factors [CRP: (SMD −1.36 [−1.75, −0.96], *P* < 0.00001); ESR: (WMD −9.09 [−11.80, −6.38], *P* < 0.00001); RF: (SMD −1.21 [−1.69, −0.73], *P* < 0.00001)]. Regarding safety, adding IGU will not increase the incidence of adverse events.

**Conclusion:**

IGU might emerge as a promising and secure therapeutic modality for addressing AS, OA, and RA.

**Systematic Review Registration:**

Identifier PROSPERO: CRD42021289249

## 1 Introduction

Arthritis encompasses various joint diseases and is associated with factors such as degenerative diseases and autoimmunity. Its hallmark features include chronic inflammation in one or more joints, often leading to pain and frequently resulting in disability. Primary clinical symptoms encompass joint pain, swelling, stiffness, and restricted mobility (Venetsanopoulou et al., 2023; Di Matteo et al., 2023). Epidemiological evidence indicates that arthritis is most prevalent among females, with an increasing incidence with age. Moreover, the prevalence of arthritis of different etiologies varies across populations (Syed et al., 2023; Katz and Bartels, 2024). Current research suggests the existence of over 100 distinct forms of arthritis, with osteoarthritis (OA) and rheumatoid arthritis (RA) being the most common; other types mainly involve arthritis linked to autoimmune diseases (Clark, 2023; Messina et al., 2023). Despite varying etiologies, these diseases are characterized by joint inflammation, resulting in pain and limited mobility (Messina et al., 2023). Presently, treatments for arthritis, both pharmacological and non-pharmacological, primarily address the progression of joint pain and the resolution of joint inflammation, especially with a common foundation in pain management (Marín et al., 2023). Osteoarthritis, a degenerative joint disease, is increasingly prevalent with the aging population (Gulati et al., 2023). According to the World Health Organization (WHO), there are over 400 million osteoarthritis patients globally (Minnig et al., 2024). In Asia, one in every six individuals is expected to develop OA at some stage (Minnig et al., 2024). Epidemiological investigations reveal that this growth is, in part, due to the rapid increase in the elderly and obese populations, resulting in a rise in osteoarthritis incidence (Wei et al., 2023; Scheuing et al., 2023; Perruccio et al., 2024). Rheumatoid arthritis (RA), characterized by primary synovial inflammation, is a chronic, disabling, autoimmune disease that can occur at any age, with a disability rate of up to 61.3% for a disease duration ≥1 year, significantly impacting patients’ physical function and quality of life (Lau, 2023; Burmester and Pope, 2017). Apart from joint pain, swelling, and restricted mobility, 40% of patients may also experience extra-articular manifestations (EAMs), among which interstitial lung disease (ILD) is a common EAM in RA and a pivotal factor contributing to the high mortality rate associated with RA (Gravallese and Firestein, 2023). RA remains challenging to cure currently; nevertheless, standardized diagnostic and therapeutic interventions can achieve optimal treatment outcomes. However, without consistent treatment, it may lead to joint deformities and functional loss (Gravallese and Firestein, 2023). Other forms of arthritis are also linked to inflammation and pain, posing significant burdens on patients, yet effective treatments addressing the root causes are still lacking.

Currently, the primary objective of arthritis treatment is to alleviate joint pain caused by arthritis inflammation, daily joint wear and tear, and muscle strains (Juma et al., 2023). Existing medications for managing arthritis encompass analgesics, steroids, non-steroidal anti-inflammatory drugs (NSAIDs), and biologic/targeted therapies aimed at alleviating severe pain and inflammation symptoms (Harmalkar et al., 2024). However, these medications entail numerous side effects that hinder their sustained ability to mitigate disease symptoms and progression over prolonged use. For instance, NSAIDs are linked to severe gastrointestinal complications and inadequate pain relief post-treatment, while biologic/targeted therapies present risks of immune disruption and adverse cardiovascular events (Di Matteo et al., 2023; Mohapatra et al., 2023; Taylor, 2023). Consequently, the treatment landscape for arthritis has evolved towards comprehensive management and therapy, with alternative modalities gradually becoming integral components of this holistic approach to management and treatment (Brown et al., 2024; Sarzi-Puttini et al., 2023). Disease-modifying antirheumatic drugs (DMARDs) serve as principal therapeutics for RA, and the emergence of novel conventional synthetic DMARDs (csDMARDs) and biologic/targeted DMARDs (b/tsDMARDs) in recent years has heralded groundbreaking advancements in the treatment of RA and RA-ILD (Brown et al., 2024; Sarzi-Puttini et al., 2023).

Iguratimod (IGU), regarded as a new type of csDMARDs, exhibits a diverse mechanism of action with comprehensive immune-regulatory effects (Ito, 2016). Studies indicate that IGU can modulate the immune balance mediated by T cells and associated inflammatory factors by regulating the quantities of helper T cells (e.g., Th1 and Th17), follicular helper T (Tfh) cells, and regulatory T (Treg) cells. Additionally, IGU can inhibit the differentiation of B cells into plasma cells, thereby suppressing the production of autoantibodies (Liu et al., 2021). In recent years, massive randomized controlled trials have been published, so there is an urgent need to summarize the efficacy and safety of IGU in treating inflammatory arthritis. This study provides future clinicians with better evidence for clinical practice, and it also offers more details for future clinical trial design by conducting a comprehensive systematic review and meta-analysis of these RCTs.

## 2 Materials and methods

### 2.1 Protocol

This systematic review and meta-analysis were conducted strictly in accordance with the protocol registered in PROSPERO (CRD42021289249) and PRISMA guidelines (see Supplementary Materials). There were not any significant deviations from the protocol.

### 2.2 Literature retrieval strategy

Chinese databases [VIP Database, China National Knowledge Infrastructure (CNKI), Wanfang Database and SINOMED] and English databases (Embase, PubMed, Medline Complete, Web of Science, Cochrane Library and ClinicalTrials.gov) were used for searching literature on IGU for the treatment of inflammatory arthritis. The retrieval period spans from the inception date to 1 February 2024. The search strategy is shown in Supplementary Table S1.

### 2.3 Search criteria

#### 2.3.1 Inclusion criteria

1) Participants: Patients diagnosed with any type of inflammatory arthritis and degenerative arthritis by accepted criteria. 2) Intervention methods: The therapeutic approach in the experimental group involved the utilization of IGU, with unrestricted parameters in terms of dosage, formulation, and administration method. 3) Control: The therapeutic regimen in the control group encompassed interventions that did not include IGU, such as placebos and conventional therapies. 4) Outcomes: Disease-related therapeutic efficacy indicators, inflammation markers, and IGU-related adverse events. 5) Study design: randomized controlled trials (RCTs).

#### 2.3.2 Exclusion criteria

1) Duplicate articles; 2) observational studies; 3) reviews, case reports, animal experiments, etc. ; 4) retracted articles.

### 2.4 Literature screening and data extraction

Initially, a preliminary literature search was conducted based on titles, abstracts, and keywords to select relevant literature initially. Subsequently, further literature inclusion was performed following established search criteria. Details regarding the study, including basic information, grouping methods, baseline conditions, treatment protocols, duration, and outcome measures, were extracted using predefined data extraction forms (Deeks et al., 2020a). Two researchers independently executed this process, with results cross-checked and any discrepancies resolved through discussion involving the entire team.

### 2.5 Risk of bias assessments

The quality assessment was conducted using the risk of bias assessment tools for RCTs recommended in the Cochrane Handbook (Deeks et al., 2020b). Each study was evaluated based on criteria, including random sequence generation, allocation concealment, blinding, attrition, and selective reporting risks. Two researchers independently performed bias risk assessments, with any inconsistencies resolved through discussion involving all researchers.

### 2.6 Data synthesis

Statistical analyses were performed using RevMan 5.4 software (Deeks et al., 2020c). Relative risk (RR) was utilized as the effect measure for dichotomous variables, while weighted mean difference (WMD) and standard mean difference (SMD) were employed for continuous variables. A 95% confidence interval (CI) was set for all analyses. Heterogeneity among results was assessed using the chi-square test, and if heterogeneity was minimal (*P* > 0.1, I^2^ < 50%), a fixed-effect model was employed for analysis; otherwise, a random-effects model was utilized.

## 3 Results

### 3.1 Literature search results

**FIGURE 1:**
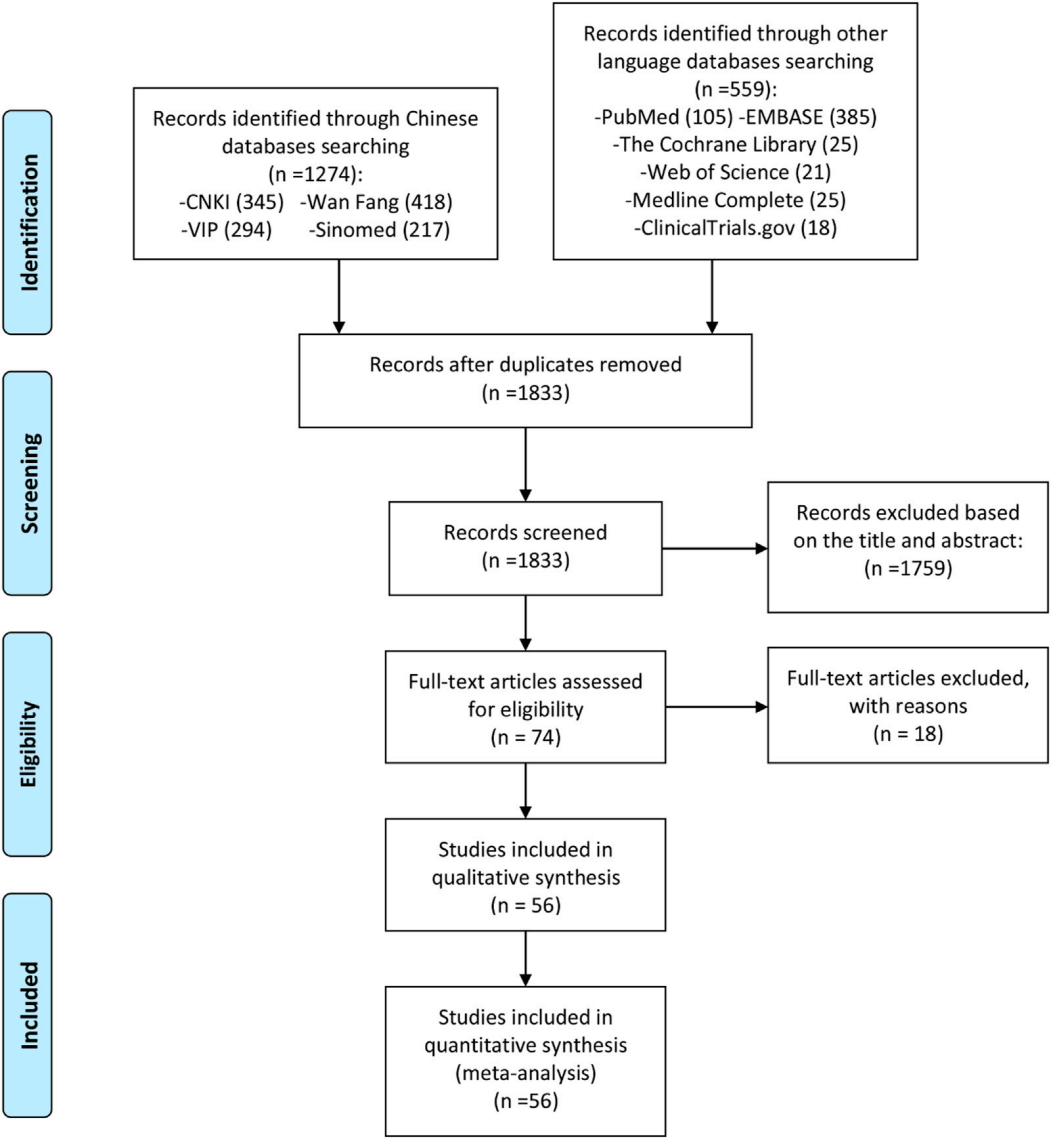
Flowchart of clinical literature searching and results screening.

A total of 1,833 initial relevant articles were identified in this study, out of which 1,759 were excluded for mismeeting the research type and content criteria. Following a thorough review of the full texts, and based on the inclusion and exclusion criteria as well as the completeness of the literature information, 18 articles were excluded for not being RCTs (Gu et al., 2020; Guifeng and Yasong, 2014; He et al., 2015; Huang and Ma, 2018; Man and Yongxin, 2020; Lin, 2016; Luo et al., 2019; Luo et al., 2018; Meng et al., 2016a; Okamura et al., 2015; Shang et al., 2019; Suto et al., 2019; Wang et al., 2017; Wang et al., 2018; Wang, 2017; Xu et al., 2021; Yoshioka et al., 2016; Zhu et al., 2016). Consequently, 56 articles were included for quantitative and qualitative analysis (Li X. et al., 2021; Bai et al., 2021; Lin YP. et al., 2019; Xu BJ. et al., 2019; Zeng et al., 2016; Li Y. et al., 2021; Yuan et al., 2020; Pang et al., 2020; Zhang, 2022; Zeng et al., 2019; Zhang et al., 2023; Han et al., 2023; Wu et al., 2022; Lü et al., 2008; Hara et al., 2007; Mo and Ma, 2015; Xiong and Guanghui, 2020; Yan et al., 2022; Yi et al., 2022; Deng JX The effect of, 2017; Tian et al., 2020; Fan et al., 2020; Xie et al., 2018; Lianju et al., 2019; Zhao and Hao, 2018; Ma et al., 2019; Zhao et al., 2016; Meng et al., 2017; Xia et al., 2016; Lu, 2014; Qi et al., 2019; Zhao et al., 2017; Hu, 2014; Chen et al., 2018; Xia et al., 2020; Tian and Tao, 2017; Xu et al., 2017; Shi et al., 2015; Wang et al., 2019; Hara et al., 2014; Ishiguro et al., 2013; RAO et al., 2014; Xu et al., 2015; Bi, 2019; Sun and Li, 2022; Yan and Wang, 2018; Meng et al., 2016b; Duan et al., 2015; Xiaong et al., 2015; Lu et al., 2009; Ju et al., 2020; Meng et al., 2015; Li et al., 2016; Li and Huang, 2020; Mo et al., 2018; Shi et al., 2023). The literature screening process and results are shown in Figure 1.

### 3.2 Description of included trials

Two articles (Xia et al., 2016; Lu, 2014) originating from the same RCT were catalogued by Xia et al. (2016), Lu (2014). Similarly, two articles (Hara et al., 2014; Ishiguro et al., 2013) derived from the same RCT were documented by Hara et al. (2014), Ishiguro et al. (2013). Consequently, the 56 records pertain to 54 RCTs. In some randomized controlled trials with two experimental groups, the control group was divided into two equal portions to match them, each representing half of the population and labelled as Group A and Group B. Detailed characteristics of the studies are presented in Supplementary Information, Supplementary Table S2.

### 3.3 Risk of bias assessments

**FIGURE 2:**
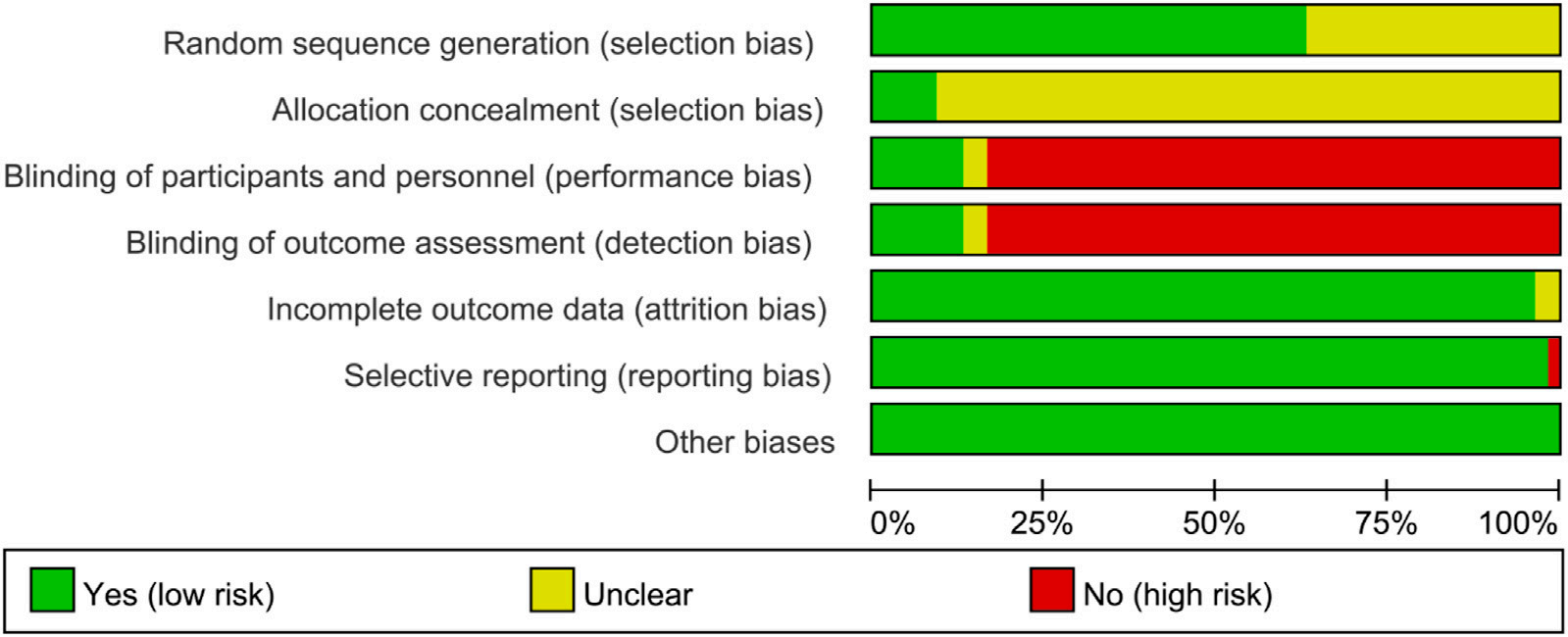
Risk of bias graph.

**FIGURE 3:**
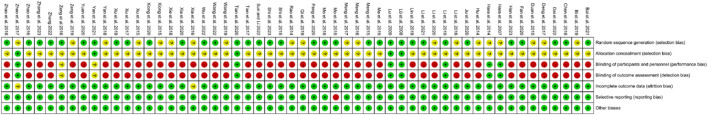
Risk of bias summary.

The graph and summary of bias risk are shown in Figures 2, 3, respectively.

### 3.4 The outcomes of IGU in the treatment of AS

#### 3.4.1 The bath ankylosing spondylitis disease activity index (BASDAI)

**FIGURE 4:**
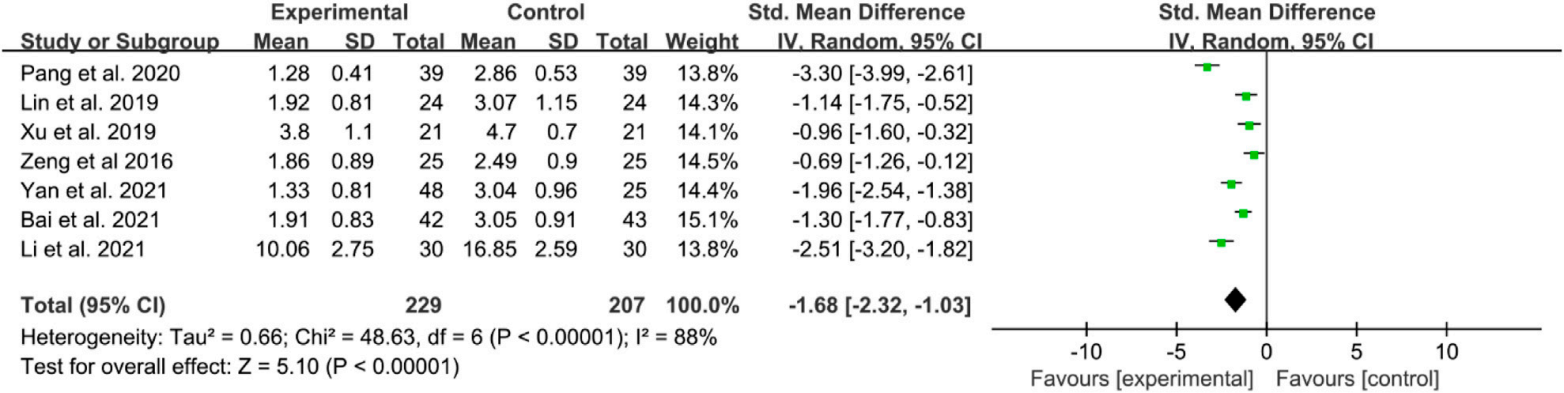
The results of BASDAI.

There are seven RCTs reporting BASDAI in their publication. The included studies showed high heterogeneity; thus, a random effects model was utilized. The IGU groups showed significantly lower BASDAI scores compared to the control groups (SMD −1.68 [−2.32, −1.03], *P* < 0.00001, Figure 4).

#### 3.4.2 Bath ankylosing spondylitis functional index (BASFI)

**FIGURE 5:**
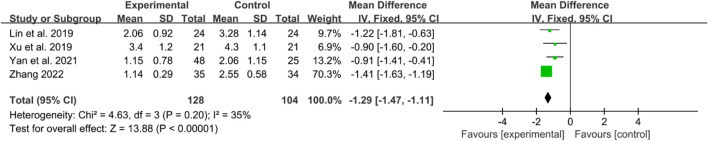
The results of BASFI.

Four RCTs reported BASFI in their manuscripts. The heterogeneity test showed low heterogeneity, a fixed effects model was used. The IGU group had a significantly lower BASFI score compared to the control group (WMD −1.29 [−1.47, −1.11], *P* < 0.00001) (Figure 5).

#### 3.4.3 Inflammatory factor

**FIGURE 6:**
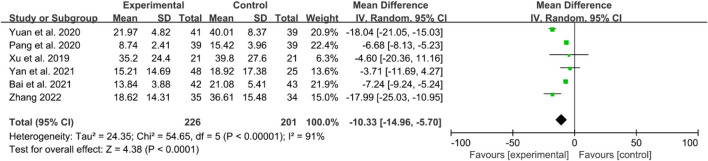
The results of ESRs.

The inflammatory factors focused on in this part of the study include erythrocyte sedimentation rates (ESRs), C-reactive protein (CRP) levels, and tumor necrosis factor- α (TNF-α) levels. Here, six RCTs reported ESRs. High heterogeneity was observed, and a random effects model was used. The IGU group had significantly lower ESRs compared to the control group (WMD −10.33 [−14.96, −5.70], *P* < 0.0001, Figure 6).

**FIGURE 7:**
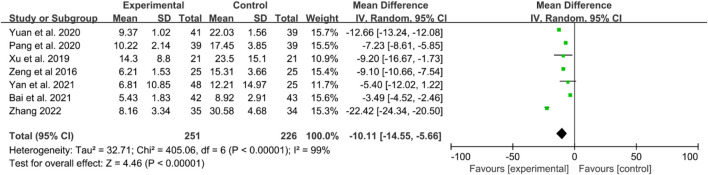
The results of CRP.

Seven RCTs reported CRP levels. The heterogeneity test indicated high heterogeneity, thus, a random effects model was utilized. The results demonstrated that IGU significantly decreased CRP levels compared to the control group (WMD −10.11 [−14.55, −5.66], *P* < 0.00001, Figure 7).

**FIGURE 8:**
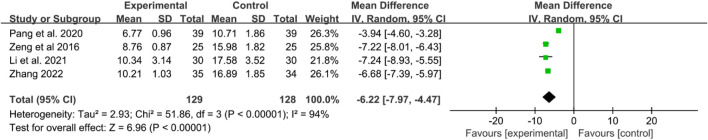
The results of TNF-α

Four RCTs reported TNF-α levels. Significant heterogeneity was detected, and a random effects model was applied. The results indicated that TNF-α levels were significantly lower in the IGU group compared to the control group (WMD −6.22 [−7.97, −4.47], *P* < 0.00001, Figure 8).

#### 3.4.4 Adverse events

Eight RCTs reported adverse events. In these RCTs, Bai et al. reported that the main adverse events were rash, abnormal liver function, and gastrointestinal reactions in 2021 (Bai et al., 2021). Lin et al. also found that in the IGU group, two cases of upper abdominal discomfort and one case of oral ulcers were observed; in contrast, the control group experienced three cases of upper abdominal discomfort, five cases of liver function abnormalities, two cases of oral ulcers, two cases of anemia, and one case of leukopenia; some patients in both groups experienced two or more adverse reactions (Lin Y. P. et al., 2019). Xu et al. reported gastrointestinal discomfort and liver function abnormalities as adverse effects (Xu B. J. et al., 2019), and Zeng et al. mainly presented gastrointestinal reactions, leukopenia, and abnormal liver function (Zeng et al., 2016). Yan et al. primarily reported gastrointestinal discomfort (Li Y. et al., 2021), while Yuan et al. showed leukopenia, oral ulcers, nausea and vomiting, diarrhea, and abnormal liver function (Yuan et al., 2020). Pang et al. briefly reported gastrointestinal reactions, abnormal liver function and rash (Pang et al., 2020). Zhang mainly showed abnormal liver and kidney function, decreased leukocytosis, and gastrointestinal discomfort (Zhang, 2022).

**FIGURE 9:**
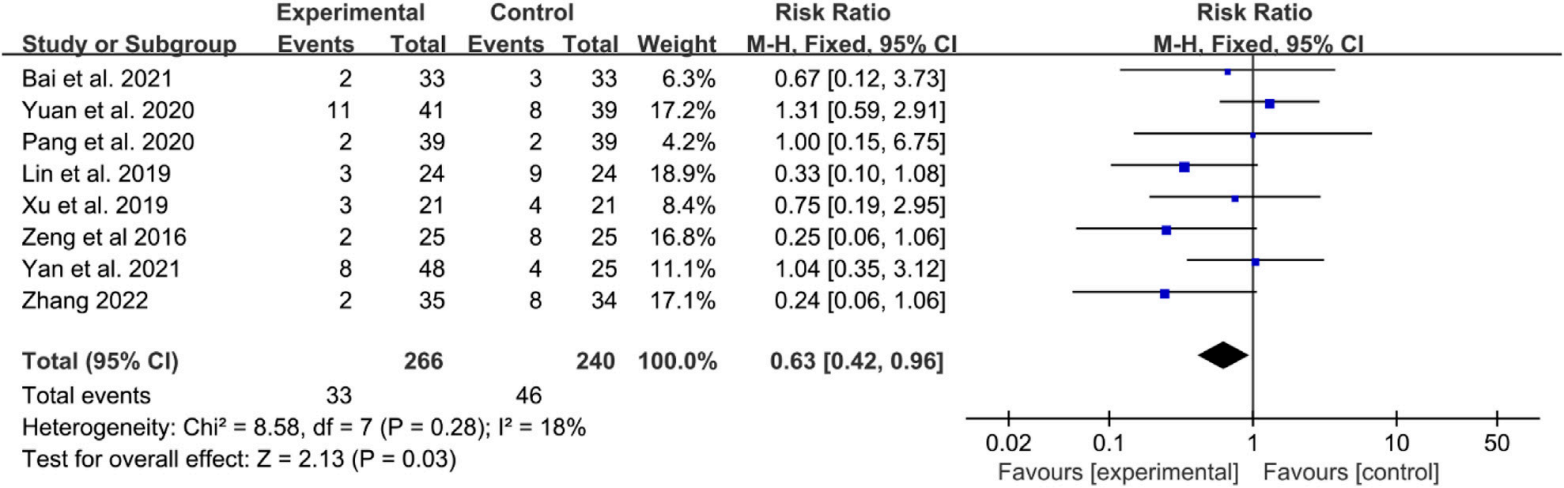
Adverse events.

The incidence rates of these adverse events were combined for meta-analysis. The heterogeneity test indicated low heterogeneity, suggesting that a fixed effects model was appropriate for analysis. The meta-analysis indicated that the incidence of adverse events in the IGU group was lower [RR 0.63 (0.24, 0.96), *P* = 0.03, Figure 9].

### 3.5 The outcomes of IGU in the treatment of OA

#### 3.5.1 Visual analog scale (VAS)

**FIGURE 10:**
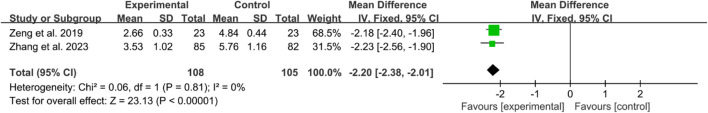
VAS.

Two RCTs reported the VAS scores of OA. The heterogeneity test indicated low heterogeneity, suggesting that a fixed effects model was appropriate for analysis. The meta-analysis revealed that the VAS in IGU group was lower (WMD −2.20 [−2.38, −2.01], *p* < 0.00001, Figure 10).

#### 3.5.2 The Western Ontario and McMaster universities osteoarthritis index (WOMAC)

**FIGURE 11:**
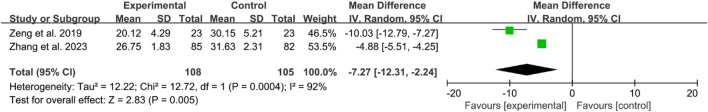
WOMAC.

Two RCTs reported WOMAC. The heterogeneity test indicated high heterogeneity, suggesting that a random effects model was appropriate for analysis. The meta-analysis indicated that the WOMAC in the IGU group was lower (WMD −7.27 [−12.31, −2.24], *P* = 0.005, Figure 11).

#### 3.5.3 Inflammation factors

The inflammatory factors in this part of the study include TNF-α and interleukin (IL)-6.

**FIGURE 12:**
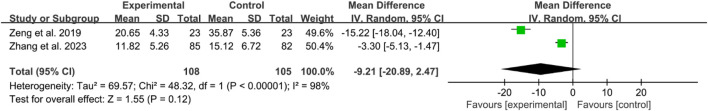
TNF-α.

Two RCTs reported TNF-α. The heterogeneity test indicated high heterogeneity, suggesting that a random effects model was appropriate for analysis. The meta-analysis indicated that the difference in TNF-α between the two groups was of no statistical significance (WMD −9.21 [−20.89, 2.47], *P* = 0.12, Figure 12).

**FIGURE 13:**
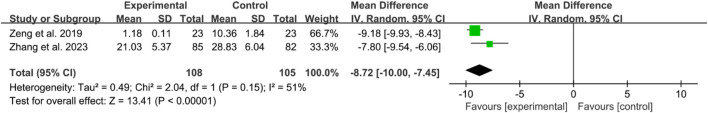
IL-6.

Two RCTs reported IL-6. The heterogeneity test indicated high heterogeneity, suggesting that a random effects model was appropriate for analysis. The meta-analysis indicated that the WOMAC in IGU group was lower (WMD −8.72 [−10.00, −7.45], *P* < 0.00001, Figure 13).

#### 3.5.4 Adverse events

In the RCT conducted by Zeng et al. in 2019, the IGU group exhibited 1 case of mild abdominal discomfort post-treatment. In contrast, the control group experienced 1 case of gastrointestinal reaction and 1 case of rash (Zeng et al., 2019). In the study by Zhang et al. (2023), it was discovered that both groups of patients did not experience any drug-related adverse reactions, indicating that the medication is relatively safe.

### 3.6 The outcomes of IGU in the treatment of RA

#### 3.6.1 RA remission rate

American College of Rheumatology (ACR)20, ACR50 and ACR70 were used to represent RA remission rate.

**FIGURE 14:**
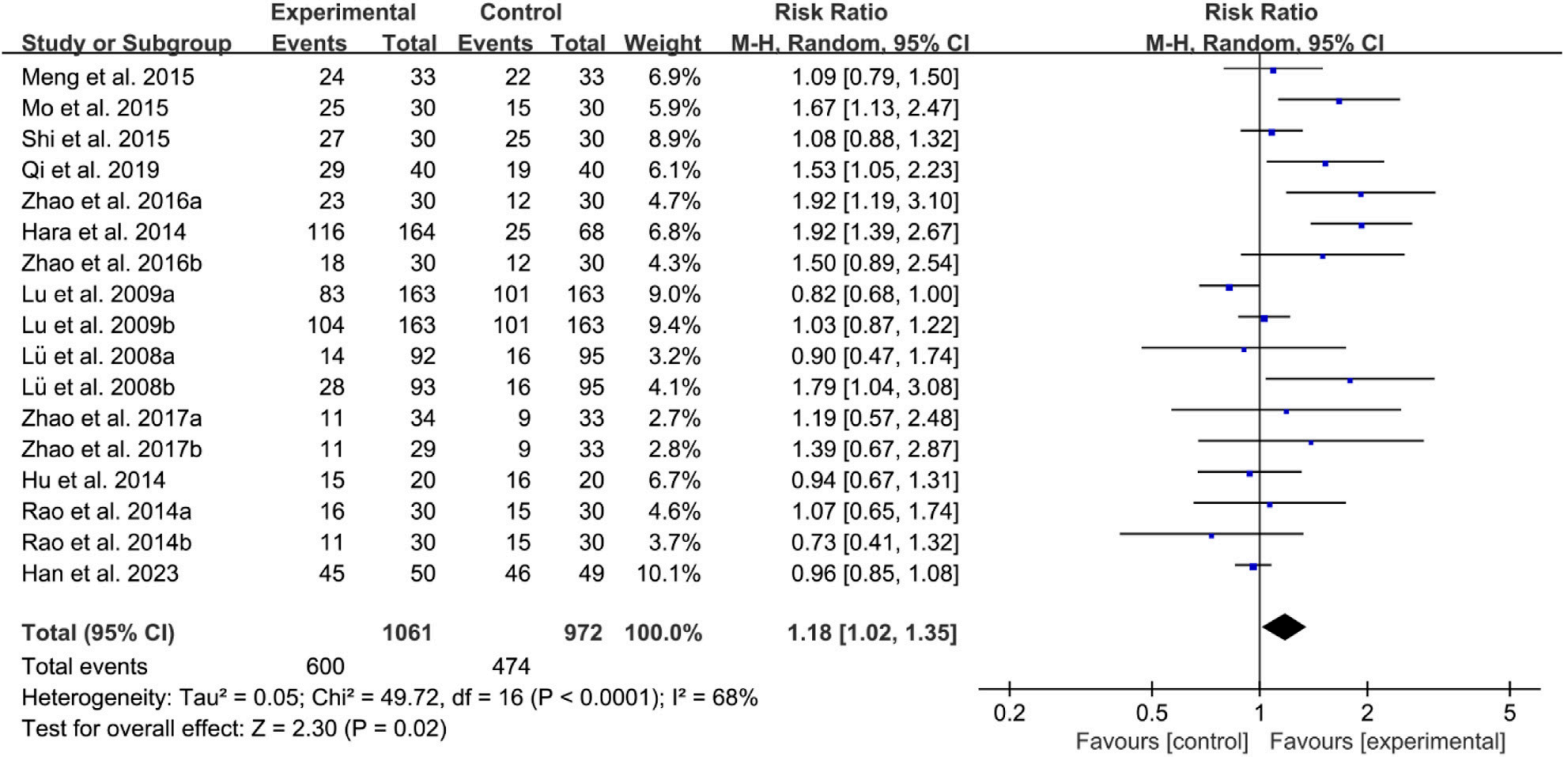
ACR20.

For ACR20, the heterogeneity test indicated high heterogeneity, suggesting that a random effects model was appropriate for analysis. The meta-analysis findings indicate that the ACR20 in the IGU group is higher than the control group (RR 1.18 [1.02, 1.35], *P* = 0.02, Figure 14).

**FIGURE 15:**
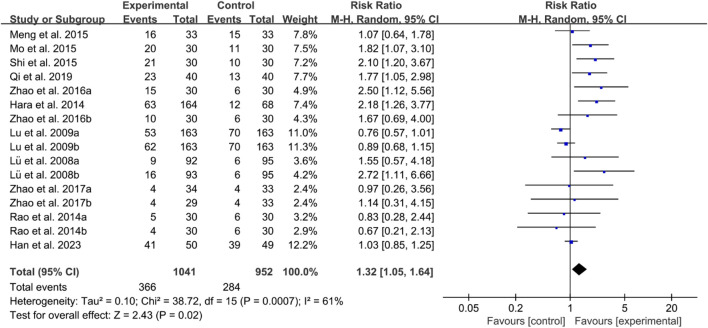
ACR50.

For ACR50, the heterogeneity test indicated high heterogeneity, suggesting that a random effects model was appropriate for analysis. The meta-analysis findings indicate that the ACR50 in the IGU group is higher than the control group (RR 1.32 [1.05, 1.64], *P* = 0.02, Figure 15).

**FIGURE 16:**
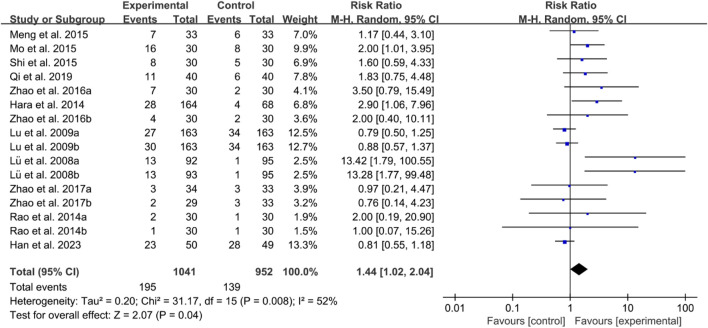
ACR70.

For ACR70, the heterogeneity test indicated high heterogeneity, suggesting that a random effects model was appropriate for analysis. The meta-analysis findings indicate that the ACR70 in the IGU group is higher than the control group (RR 1.44 [1.02, 2.04], *P* = 0.04, Figure 16).

#### 3.6.2 Disease activity score 28 (DAS28)

**FIGURE 17:**
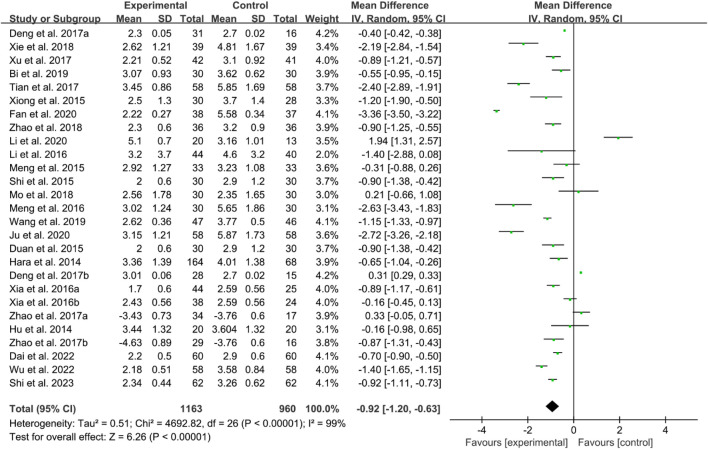
DAS28.

Twenty-four RCTs reported DAS28. The heterogeneity test indicated high heterogeneity, suggesting that a random effects model was appropriate for analysis. The meta-analysis findings indicate that the DAS28 in IGU group is lower than control group (WMD −0.92 [−1.20, −0.63], *P* < 0.00001, Figure 17).

#### 3.6.3 Inflammatory factor

Inflammatory factors focused in this section include CRP, ESR and rheumatoid factor (RF).

**FIGURE 18:**
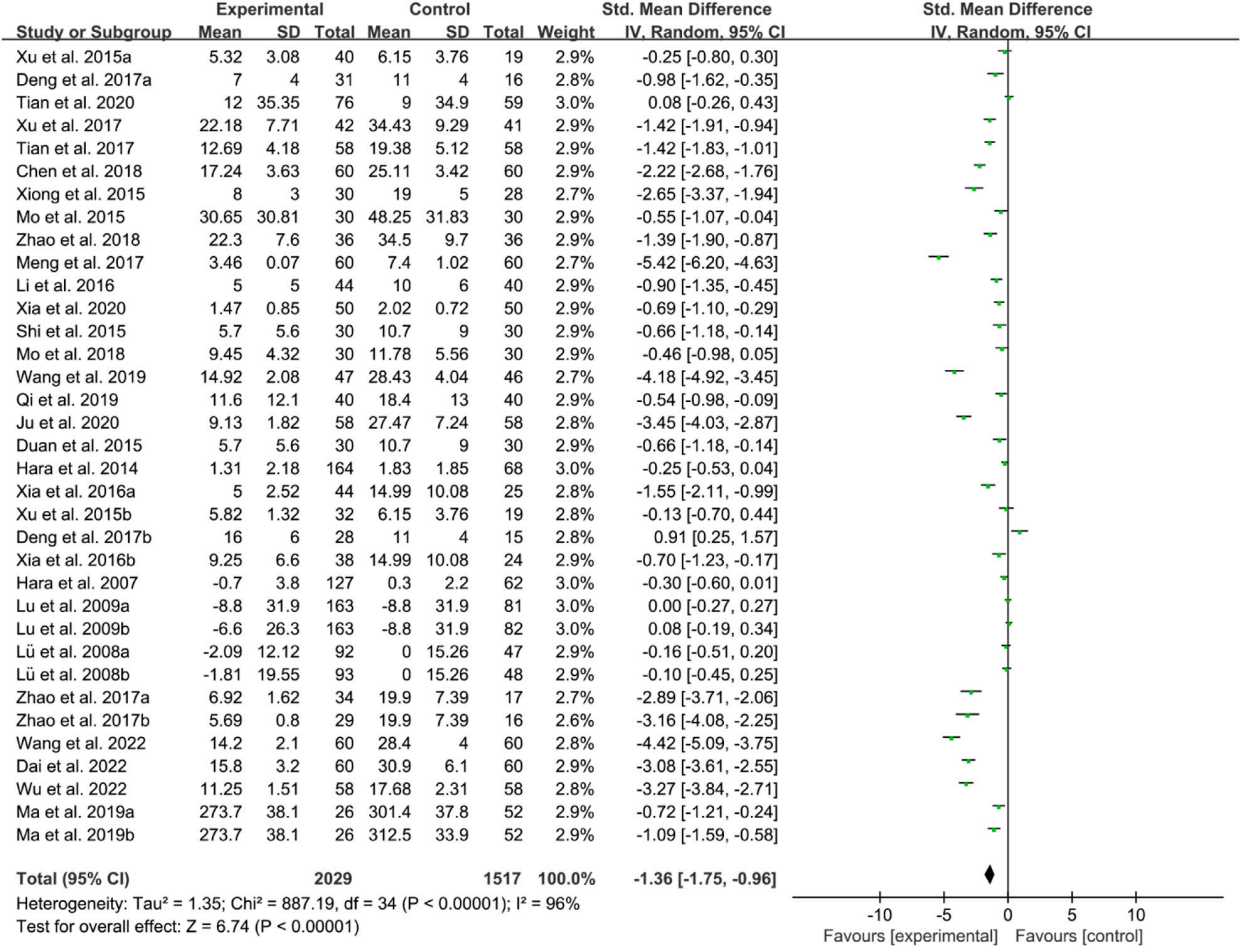
CRP.

For CRP, the heterogeneity test indicated high heterogeneity, suggesting that a random effects model was appropriate for analysis. The meta-analysis findings indicate that the CRP in the IGU group is higher than the control group (SMD −1.36 [−1.75, −0.96], *P* < 0.00001, Figure 18).

**FIGURE 19:**
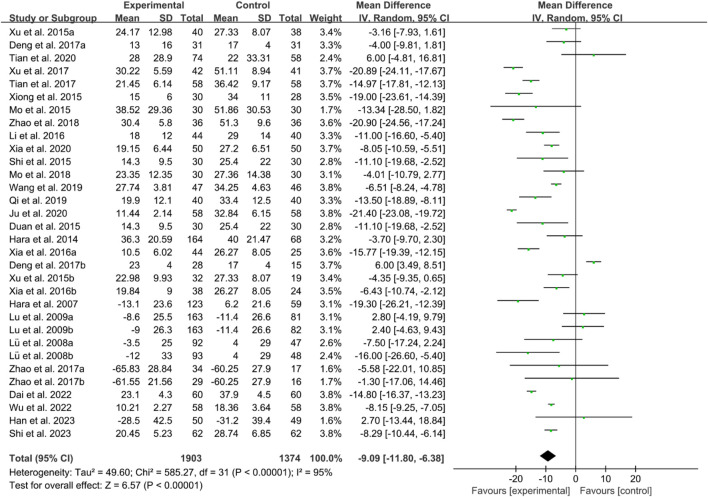
ESR.

For ESR, the heterogeneity test indicated high heterogeneity, suggesting that a random effects model was appropriate for analysis. The meta-analysis findings indicate that the ESR in the IGU group is higher than the control group (WMD −9.09 [−11.80, −6.38], *P* < 0.00001, Figure 19).

**FIGURE 20:**
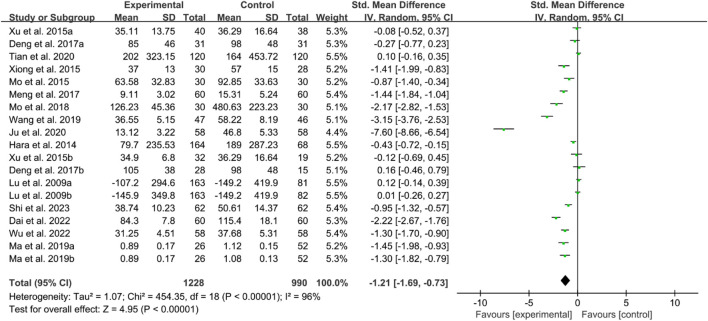
RF.

For RF, the heterogeneity test indicated high heterogeneity, suggesting that a random effects model was appropriate for analysis. The meta-analysis findings indicate that the RF in the IGU group is higher than the control group (SMD −1.21 [−1.69, −0.73], *P* < 0.00001, Figure 20).

#### 3.6.4 Adverse events

**FIGURE 21:**
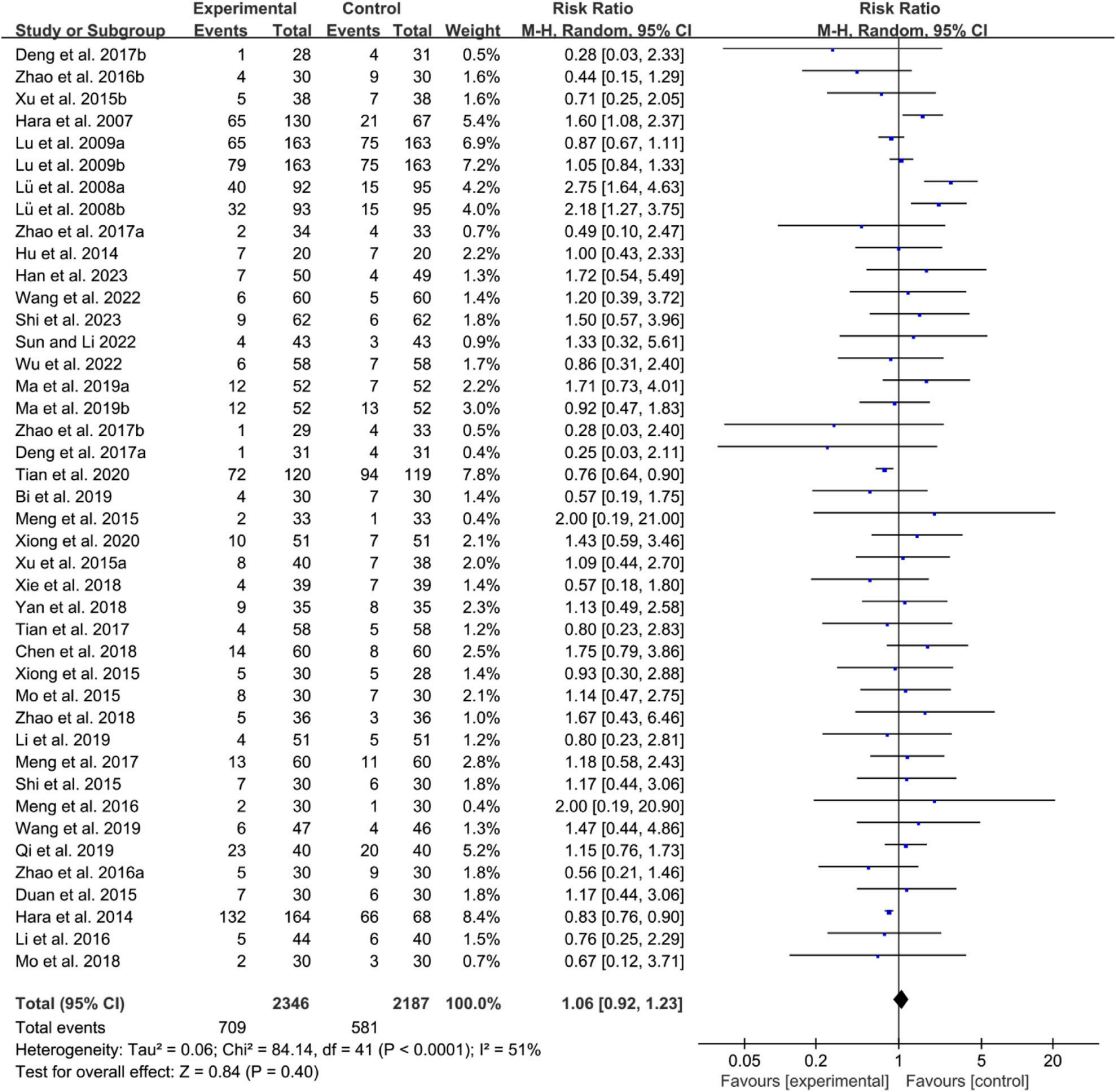
Adverse events for RA.

Thirty-five RCTs reported adverse events. The heterogeneity test indicated low heterogeneity with *P* < 0.0001 and *I* ^2^ = 51%, suggesting that a fixed effects model was appropriate for analysis. The meta-analysis showed that the incidence of adverse events between the two groups was of no statistical significance (RR 1.06 [0.92, 1.23], *P* = 0.40, Figure 21).

## 4 Discussion

### 4.1 The molecular mechanism of IGU in treating inflammatory arthritis

IGU is a novel DMARD that offers anti-inflammatory, antifibrotic, anti-resorptive, immunomodulatory, and bone metabolism-regulating effects (Shen et al., 2024; Zeng et al., 2023; Zou et al., 2023). As research on IGU has advanced in recent years, its therapeutic applications have broadened. Current evidence indicates that IGU provides significant immunomodulatory benefits and comprehensive bone protection, balancing efficacy and safety, making it well-suited for combination therapy and long-term maintenance in the treatment of rheumatoid arthritis (Hu et al., 2024; Sun et al., 2023; Long et al., 2023). Compared to traditional DMARDs, IGU has been shown to reduce rheumatoid factor significantly and anti-CCP antibody levels, effectively preventing bone destruction and reducing the risk of disability and deformity associated with arthritis (Hu et al., 2024; Sun et al., 2023; Long et al., 2023).

**FIGURE 22:**
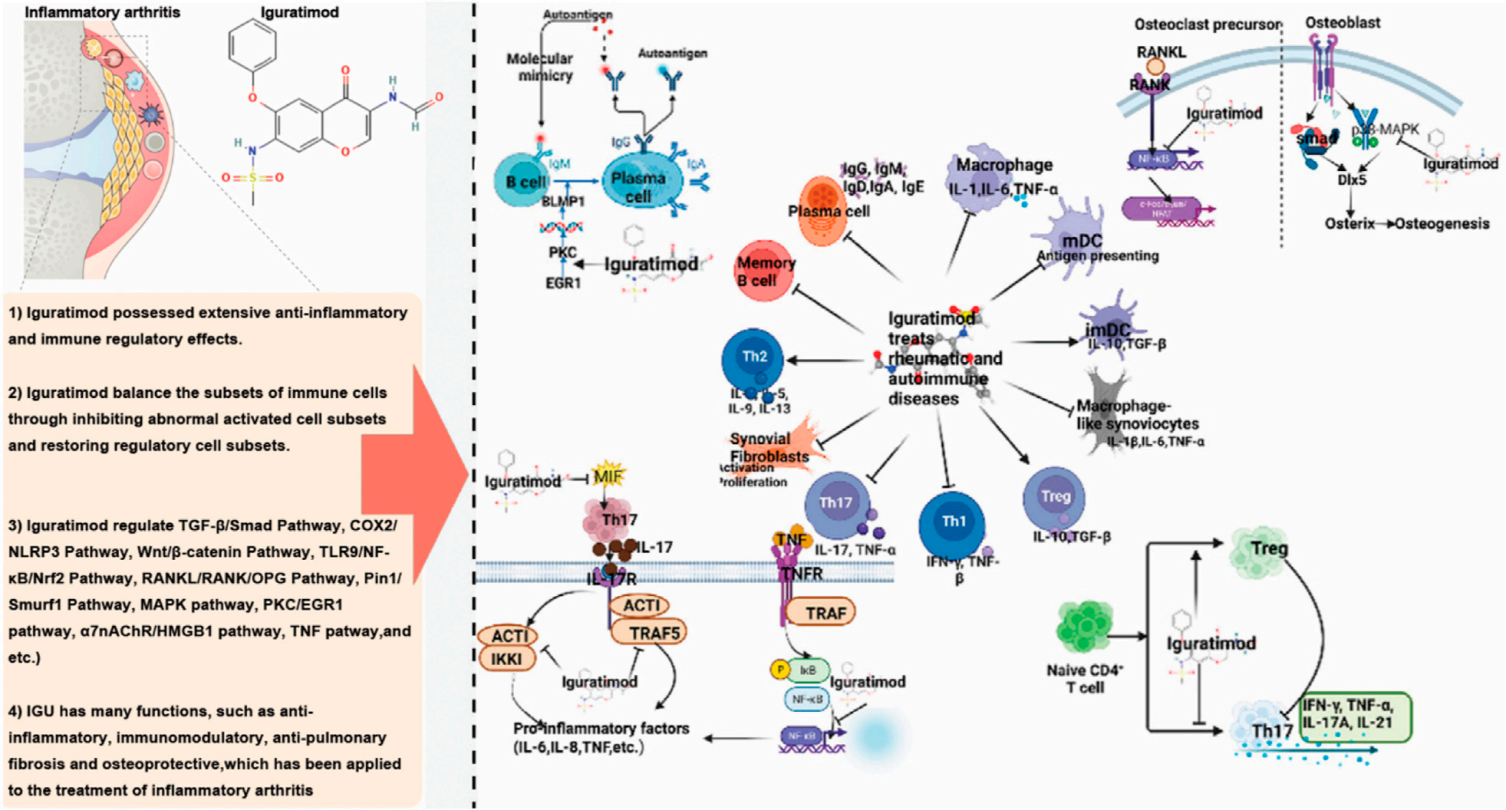
The molecular mechanism of IGU in treating inflammatory arthritis.

Regarding its anti-inflammatory effect, IGU exerts its anti-inflammatory effects by inhibiting the proliferation of inflammatory cells and reducing the release of pro-inflammatory cytokines, thereby mediating key anti-inflammatory signaling pathways (Figure 22). Specifically, IGU at lower concentrations primarily inhibits the migration of fibroblast-like synoviocytes (FLS), with higher concentrations leading to the suppression of FLS proliferation and even inducing apoptosis. In animal models of RA, OA and AS, IGU significantly reduces the expression of pro-inflammatory cytokines while increasing the expression of anti-inflammatory cytokines. This dual modulation reduces the infiltration of inflammatory cells in the bloodstream and affected tissues, enhancing its anti-inflammatory effect. In addition, IGU protects against inflammatory arthritis by activating the nuclear factor-κB (NF-κB) signaling pathway and downregulating sodium bicarbonate cotransporter e2 (NBCe2) in RA patients to inhibit protein citrullination and inflammation. In both acute and chronic inflammation models, such as carrageenan-induced paw edema and adjuvant-induced arthritis in rats, IGU demonstrated anti-inflammatory and analgesic effects. Unlike traditional NSAIDs, IGU does not cause gastrointestinal ulcer-like side effects in fasting rats and can inhibit kininogen in kaolin-induced inflammatory responses (Peng et al., 2024). IGU significantly reduces IgM production in mouse B cell cultures and promotes the switch to IgG1 under lipopolysaccharide and/or IL-4 induction (Chen et al., 2023; Nozaki, 2021). In human multiple myeloma cell cultures (ARH-77 cell line), IGU inhibits spontaneous IgG antibody production without affecting cell proliferation, and in human peripheral blood B cells induced by autologous T cells and anti-CD3 antibodies, IGU suppresses the production of both IgM and IgG, effectively inhibiting immunoglobulin production in B cells without causing blockage (Zeng et al., 2022a).

A study found that in chronic rheumatoid arthritis models, such as adjuvant-induced arthritis (AIA) rats and Murphy Roths large lymphoproliferation (MRL/lpr) mice, IGU not only improved arthritis lesions but also reduced hypergammaglobulinemia (Jiang et al., 2020). Regarding cytokine inhibition, IGU suppressed the production of IL-1β, TNF-α, IL-6, IL-8, and monocyte chemoattractant protein (MCP)-1. In synovial cell cultures derived from patients with rheumatic diseases, IGU significantly reduced the production of IL-6, IL-8, and colony-stimulating factors. Additionally, IGU inhibited the upregulation of costimulatory molecules, including CD54, CD58, and CD106, in synovial cells stimulated by IFN-γ. In a mouse model of subcutaneous air pouch inflammation, oral administration of IGU at doses of 30–100 mg/kg significantly suppressed TNFα-induced MCP-1 production (Jiang et al., 2020; Li et al., 2019). Pro-inflammatory cytokines such as IL-1, IL-6, and TNF-α play a crucial role in bone resorption and are key pathological factors in RA, closely associated with disease activity (Xie et al., 2020). These cytokines activate osteoclasts, increasing bone resorption and subsequent loss. Inhibiting IL-1, IL-6, and TNF-α can effectively control RA and prevent related bone degradation. MRI results in the collagen-induced arthritis (CIA) rat model showed that IGU nearly completely suppressed inflammation and bone marrow edema associated with CIA. X-ray and CT scans revealed significant inhibition of bone resorption and joint destruction in rats treated with IGU (Zeng et al., 2022b).

IGU also plays a significant role in promoting bone formation and regulating bone metabolism. *In vitro* studies have shown that IGU enhances osteoblast differentiation. At the same time, *in vivo* experiments demonstrate its ability to augment bone morphogenetic protein (BMP)-2-mediated bone formation, which is believed to be associated with increased Osterix (Osx) expression. Osx is crucial for bone differentiation and formation (Sun et al., 2023). Recent research has revealed that IGU improves disuse osteoporosis in mice by inhibiting sclerostin and the receptor activator of NF-κB ligand (RANKL) through the extracellular signal-regulated kinase/early growth response protein 1/TNF-α pathway in osteocytes (Miura et al., 2024). In the ST2 bone marrow stromal cell line, Osx expression is minimal in the absence of recombinant human BMP (rhBMP)-2, but IGU can stimulate Osx expression by more than threefold when rhBMP-2 is present. In the pre-osteoblast cell line MC3T3-E1, IGU directly stimulates Osx expression, independent of rhBMP-2, thereby promoting osteoblast differentiation. Further studies have shown that IGU dose-dependently stimulates the secretion of osteocalcin in both ST2 and MC3T3-E1 cells; in the presence of rhBMP-2, IGU increases calcium content in ST2 cells by 14-fold, leading to the formation of mineralized nodules. In mouse models, IGU increased calcium content in the ossicles by 1.7-fold (Hou et al., 2021).

### 4.2 IGU in the treatment of AS

AS is a chronic inflammatory disease that affects the spine and joints, and its pathogenesis is still not entirely understood. Current understanding suggests that AS results from interactions among genes, microbes, and other factors, leading to an imbalance where osteogenesis by osteoblasts surpasses bone resorption by osteoclasts, ultimately culminating in joint ankylosis (Rodolfi et al., 2024). Throughout the inflammatory process of AS, cellular factors like TNF-α and IL-1 play pivotal roles (Liu et al., 2023). Current guidelines for AS treatment still recommend the use of NSAIDs and TNF antagonists, while drugs like sulfasalazine (SSZ) and methotrexate (MTX) are recommended for those with peripheral joint involvement (Mysler et al., 2024; van de Sande and Elewaut, 2023). Studies have shown that IGU can inhibit the production of inflammatory cytokines such as interleukin-1 (IL-1), IL-6, IL-17, and TNF-α. Here, TNF-α is a crucial inflammatory factor in the pathogenesis of AS, and the IL-23/IL-17 pathway has been proven to be important in the mechanism underlying AS (Li et al., 2020; Ishikawa and Ishikawa, 2019; Macleod et al., 2023). Consequently, in recent years, multiple studies have been progressively examining the efficacy of IGU in treating AS.

This systematic review and meta-analysis showed that IGU can reduce disease activity (reduce BASDAI and BASFI) and improve patients’ inflammatory response (reduce ESR, CRP and TNF-α) in patients with AS. In terms of safety, compared with the control group, the incidence of adverse events with the addition of IGU was lower. However, given the high risk of bias in blinding and the unknown risks associated with allocation concealment and random sequence generation in most RCTs, the stability of the conclusions may be compromised. Therefore, the findings should be interpreted with caution.

### 4.3 IGU in the treatment of OA

OA is a disease that affects all joints. The increasing prevalence of OA is attributed to the accelerated aging of the population, escalating rates of obesity, and subsequent joint injuries (Knights et al., 2023; Kim, 2022). Furthermore, recent studies indicate a trend towards a younger age of onset for OA. In the early stages of OA, the primary manifestation is joint pain during activity, with relief experienced at rest. As the disease progresses, continuous pain may develop, potentially leading to joint deformity, impairing joint function, and, in severe cases, resulting in disability (Zhou et al., 2024). Research has confirmed that IGU can inhibit the production of inflammatory factors such as IL-17, TNF-α, and IL-6, exhibiting anti-inflammatory effects, while simultaneously acting as an NSAID. The mechanism of action of this drug aligns closely with the therapeutic goals of treating OA (Horváth et al., 2023). It has been found that IGU has clear chondroprotective effects in rheumatoid arthritis currently (Cong et al., 2021), and new research similarly suggests that IGU assists in protecting cartilage in OA (Xu B. et al., 2019; Xu et al., 2023). Studies have shown that in rats with IGU administered orally, there is an increase in the expression of transient receptor potential cation channel subfamily V member 4 (TRPV4) in cartilage, resulting in significant pain reduction and notable inhibition of cartilage destruction. Following *in vitro* experiments involving the cultivation of cartilage cells post-IGU intervention, it is observed that in rats receiving IGU treatment, the differentiation, activity, and migratory capabilities of rat cartilage cells are significantly enhanced. Hence, based on preliminary results, IGU appears to delay cartilage degradation and promote differentiation and migration, possibly acting through the TRPV4 ion channel (Xu et al., 2023).

This systematic review and meta-analysis showed that IGU can reduce pain caused by OA (reduce VAS and WOMAC) and improve patients’ inflammatory response (reduce IL-6). Regarding safety, adding IGU does not increase the incidence of adverse events. However, as the number of RCTs was only two and there was a high risk of bias in blinding and an unknown risk of bias in allocation concealment, the conclusions need to be interpreted with caution.

### 4.4 IGU in the treatment of RA

RA is an autoimmune disease primarily characterized by symmetrical damage to small joints. Research indicates that IGU can selectively inhibit cyclooxygenase-2 (COX-2) and NF-κB to alleviate inflammatory responses, particularly in cases of primary or secondary drug resistance in RA (Deng et al., 2022). IGU primarily functions by suppressing inflammatory cytokines to inhibit the occurrence and progression of synovitis. Recent studies demonstrate that IGU can effectively restrain the proliferation of RA-FLS. Wang et al. substantiated that IGU can selectively repress the expression of COX-2 mRNA and c-fos mRNA, subsequently inhibiting RA-FLS proliferation, with the inhibitory effects conforming to a dose-response relationship (WANG and SHEN, 2015). Du et al. revealed that IGU decreases the expression of matrix metalloproteinase (MMP)-1 and MMP-3, thereby suppressing excessive proliferation of FLS (Du et al., 2012). Additionally, Meng et al. demonstrated that IGU reduces vascular endothelial growth factor release, enhances endothelin production, and reduces synovial vascular neogenesis consequently (Meng D. Z. et al., 2016).

Lin J. et al. (2019) showed that through cell migration experiments, IGU significantly inhibits the invasive behavior of RA-FLS via the mitogen-activated protein kinase (MAPK) signaling pathway and promotes apoptosis. Pathological changes in bone loss in RA joints are closely associated with the activation of pro-inflammatory factors leading to osteoclast activation and bone resorption. Osteoprotegerin (OPG) competes with the receptor activator of NF-κB ligand (RANKL) for binding to the activator of NF-κB receptor (RANK). Clinical studies illustrate that IGU can lower serum IL-17 levels to attenuate the expression of inflammatory factors like IL-9 and IL-8, reduce RANKL levels, and directly modulate the OPG/RANKL/RANK axis system, consequently delaying bone destruction. Combining with MTX can significantly increase OPG levels, yielding better therapeutic outcomes (Luo et al., 2013). Feng et al. indicated that IGU plays a pivotal role in inhibiting the expression of genes essential for osteoclast differentiation and activation, such as RANK, acidic phosphatase, tissue protease K, and MMP-1, thereby inhibiting osteoclast proliferation and differentiation, and showing a dose-dependent relationship with efficacy (Feng et al., 2019). Positive anti-citrullinated protein antibody status is closely associated with bone loss in RA, and IGU can dose-dependently downregulate peptidyl arginine deiminase (PADI) 2 and PADI4 in neutrophils, thereby suppressing protein citrullination and alleviating bone loss (Li et al., 2020).

MTX serves as an anchor drug for treating RA and is commonly used in combination with IGU. A meta-analysis by Shrestha et al. suggested that at 24 weeks, the therapeutic effects, disease status, and adverse reactions exhibited by IGU and MTX are similar, indicating the potential of IGU as a substitute for MTX (Shrestha et al., 2020). Additionally, another meta-analysis by Wu et al. revealed that the combination of IGU and MTX in the treatment of RA leads to superior efficacy in increasing ACR20/50/70 response rates, reducing ESR, CRP, assessing the activity of 28 joint diseases, and VAS scores compared to individual use, without a significant increase in adverse reactions (Wu et al., 2018). A study by Ren et al. (2017) suggested that combination therapy can significantly reduce abnormally elevated platelet counts and decrease serum immunoglobulins (IgA, IgG, IgM) and T lymphocyte subsets (CD3+, CD4+ and CD8+ T cells).

The lungs are one of the most frequently affected extra-articular organs in RA, primarily manifesting as interstitial lung disease. Prolonged, low-dose use of MTX can lead to adverse reactions, causing interstitial lung disease. Han et al. found in animal experiments that IGU improves bleomycin-induced spontaneous pulmonary fibrosis by suppressing inflammation (Han et al., 2018). Short-term clinical observations by Hao et al. indicate that IGU effectively treats RA combined with chronic interstitial pneumonia, with a lower incidence of adverse reactions and no concomitant infections (L and i, 2014). Zhao et al. demonstrated that IGU can ameliorate lung tissue fibrosis by inhibiting the expression of factors like MMP-9, IL-1, and IL-6 (Zhao et al., 2019). Therefore, in patients with lung complications, consider prioritizing IGU. RA specifically impacts the cardiovascular system, including the cardiac conduction system. An essential factor contributing to heart function impairment in RA patients is the imbalance between oxidation and the antioxidant system. A clinical study on refractory RA revealed that the combination of IGU and MTX can increase superoxide dismutase, reduce total antioxidant capacity, and, in controlling oxidative stress, suppress cardiovascular diseases associated with RA (Lai, 2018).

This systematic review and meta-analysis showed that IGU can reduce disease activity (increase RA remission rate and reduce DAS28) and improve patients’ inflammatory response (reduce ESR, CRP and RF) in patients with RA. Regarding safety, adding IGU does not increase the incidence of adverse events. However, considering that most RCTs have a high risk of bias in blind implementation and an unknown risk of bias in allocation concealment and random sequence generation, especially Mo et al. (2018), which has a high risk of bias in selective reporting, the stability of the conclusions has been affected to a certain extent, and the conclusions need to be interpreted with caution.

### 4.5 Possible sources of heterogeneity

The heterogeneity of BASDAI, WOMAC, ACR and some inflammatory factors was high. We consider that this may be related to the following reasons: 1) basic characteristics of patients (such as age, gender, severity of disease, etc.); 2) different IGU preparations, or different combination therapies or basic treatments for each patient and RCT; 3) The heterogeneity of subjective outcome indicators (BASDAI, WOMAC and ACR) may be related to the high risk of bias of the blinding method.

### 4.6 Limitations and future prospects

This systematic review and meta-analysis has several limitations. First, most of the included RCTs were conducted in China and Japan, which may limit the applicability of the findings to the East Asian populations. As a result, the conclusions drawn from this analysis may primarily reflect the effectiveness of IGU in treating RA, AS, and OA in East Asian individuals. Additionally, the limited number of RCTs focusing on IGU for OA patients underscores the need for more studies to strengthen the evidence base. Furthermore, since IGU has only been recently introduced, its mechanisms of action and interactions with other medications, such as MTX and leflunomide, require further exploration. In summary, IGU demonstrates superior efficacy in treating RA, OA, and AS compared to control groups without increasing the incidence of adverse reactions. This suggests that IGU offers a promising new treatment option for these conditions. However, further multicenter, large-sample, high-quality randomized controlled trials are needed to provide more robust evidence.

## 5 Conclusion

Given the existing data, IGU might emerge as a promising and secure therapeutic modality for addressing AS, OA, and RA. Nevertheless, additional RCTs are imperative to assess its effectiveness across other inflammatory joint disorders.

## Data Availability

The original contributions presented in the study are included in the article/Supplementary Material, further inquiries can be directed to the corresponding authors.
