# Supplementary material for: A systematic review and meta-analysis of the efficacy and safety of iguratimod in the treatment of inflammatory arthritis and degenerative arthritis
Source: Front Pharmacol. 2024 Oct 10;15:1440584. doi: 10.3389/fphar.2024.1440584 (PMC11499590; doi:10.3389/fphar.2024.1440584)
Supplement: Supplementary file 1 [file Table1.docx]

**Table S1.** Search Strategies for Pubmed and Embase

| **PubMed** | (Iguratimod OR Alamode OR T-614 OR C17H14N2O6S OR CAS 123663-49-0 OR IGU OR 3-Formylamino-7-methylsulfonylamino-6-phenoxy-4H-1-benzopyran-4-one)  AND  ((Arthritis OR Arthritides OR Polyarthritis OR Polyarthritides) OR (Psoriasis OR Psoriases OR Pustulosis of Palms and Soles OR Pustulosis Palmaris et Plantaris OR Palmoplantaris Pustulosis OR Pustular Psoriasis of Palms and Soles) OR (hyperuricemi* OR hyperuricaemi* OR hyperuricacid* OR Gout OR Gouts) OR (Osteoporoses OR Osteoporosis, Post-Traumatic OR Osteoporosis, Post Traumatic OR Post-Traumatic Osteoporoses OR Post-Traumatic Osteoporosis OR Osteoporosis, Senile OR Osteoporoses, Senile OR Senile Osteoporoses OR Osteoporosis, Involutional OR Senile Osteoporosis OR Osteoporosis, Age-Related OR Osteoporosis, Age Related OR Bone Loss, Age-Related OR Age-Related Bone Loss OR Age-Related Bone Losses OR Bone Loss, Age Related OR Bone Losses, Age-Related OR Age-Related Osteoporosis OR Age Related Osteoporosis OR Age-Related Osteoporoses OR Osteoporoses, Age-Related OR Metabolic Bone Diseases OR Bone Disease, Metabolic OR Metabolic Bone Disease OR Osteopenia OR Osteopenias OR Low Bone Density OR Bone Density, Low OR Low Bone Densities OR Low Bone Mineral Density) OR (Rheumatoid arthritis OR Arthritis, Rheumatoid) OR (Osteoarthritis OR Osteoarthritides OR Osteoarthrosis OR Osteoarthroses OR Arthritis, Degenerative OR Arthritides, Degenerative OR Degenerative Arthritides OR Degenerative Arthritis OR Osteoarthrosis Deformans) OR (Spondyloarthritis Ankylopoietica OR Ankylosing Spondylarthritis OR Ankylosing Spondylarthritides OR Spondylarthritides, Ankylosing OR Spondylarthritis, Ankylosing OR Ankylosing Spondylitis OR Spondylarthritis Ankylopoietica OR Bechterew Disease OR Bechterew's Disease OR Bechterews Disease OR Marie-Struempell Disease OR Marie Struempell Disease OR Rheumatoid Spondylitis OR Spondylitis, Rheumatoid OR Spondylitis Ankylopoietica OR Ankylosing Spondyloarthritis OR Ankylosing Spondyloarthritides OR Spondyloarthritides, Ankylosing OR Spondyloarthritis, Ankylosing) OR (Juvenile Arthritis OR Arthritis, Juvenile Chronic OR Chronic Arthritis, Juvenile OR Juvenile Rheumatoid Arthritis OR Arthritis, Juvenile Idiopathic OR Juvenile Chronic Arthritis OR Arthritis, Juvenile Rheumatoid OR Rheumatoid Arthritis, Juvenile OR Juvenile Idiopathic Arthritis OR Idiopathic Arthritis, Juvenile OR Juvenile-Onset Still Disease OR Juvenile Onset Still Disease OR Still's Disease, Juvenile-Onset OR Juvenile-Onset Still's Disease OR Still's Disease, Juvenile Onset OR Still Disease, Juvenile-Onset OR Still Disease, Juvenile Onset OR Systemic Arthritis, Juvenile OR Arthritis, Juvenile Systemic OR Juvenile Systemic Arthritis OR Juvenile-Onset Stills Disease OR Juvenile Onset Stills Disease OR Stills Disease, Juvenile-Onset OR Polyarthritis, Juvenile, Rheumatoid Factor Positive OR Polyarthritis, Juvenile, Rheumatoid Factor Negative OR Oligoarthritis, Juvenile OR Juvenile Oligoarthritis OR Enthesitis-Related Arthritis, Juvenile OR Arthritis, Juvenile Enthesitis-Related OR Enthesitis Related Arthritis, Juvenile OR Juvenile Enthesitis-Related Arthritis OR Psoriatic Arthritis, Juvenile OR Arthritis, Juvenile Psoriatic))  AND  (random* controlled trial [pt] OR controlled clinical trial* [pt] OR randomized [tiab] OR placebo [tiab] OR drug therapy [sh] OR random* [tiab] OR trial* [tiab] OR group* [tiab])  NOT  (animals [mh] NOT humans [mh]) |
| --- | --- |
| **EMBASE** | 1 Osteoporoses/exp  2 Post-Traumatic Osteoporoses or Post-Traumatic Osteoporosis  3 Senile Osteoporoses or Senile Osteoporosis  4 Age-Related Bone Loss or Age-Related Bone Losses  5 Age-Related Osteoporosis or Age Related Osteoporosis or Age-Related Osteoporoses  6 Osteoarthritis/exp  7 Osteoarthritides/  8 Osteoarthrosis/  9 Osteoarthroses/  10 Degenerative Arthritides/  11 Degenerative Arthritis/  12 Osteoarthrosis Deformans/  13 Spondyloarthritis Ankylopoietica/  14 Ankylosing Spondylarthritis/  15 Ankylosing Spondylarthritides/  16 Ankylosing Spondylitis/  17 Spondylarthritis Ankylopoietica/  18 Bechterew Disease/  19 Bechterews Disease/  20 Marie-Struempell Disease/  21 Marie Struempell Disease/  22 Rheumatoid Spondylitis/  23 Spondylitis Ankylopoietica/  24 Ankylosing Spondyloarthritis/  25 Ankylosing Spondyloarthritides/  26 Arthritis, rheumatoid/exp  27 Rheumatoid arthritis/  28 Arthritis, Juvenile/exp  29 Juvenile Arthritis/  30 Juvenile Rheumatoid Arthritis/  31 Juvenile Chronic Arthritis/  32 Juvenile Idiopathic Arthritis/  33 Juvenile-Onset Still Disease/  34 Juvenile Onset Still Disease/  35 Juvenile Systemic Arthritis/  36 Juvenile-Onset Stills Disease/  37 Juvenile Onset Stills Disease/  38 Juvenile Oligoarthritis/  39 Arthritis, Gouty/exp  40 Gouty Arthritis/  41 Gouty Arthritides/  42 Arthritides, Reactive/exp  43 Reactive Arthritides/  44 Reactive Arthritis/  45 Post-Infectious Arthritides/  46 Post-Infectious Arthritis/  47 Post Infectious Arthritis/  48 Postinfectious Arthritis/  49 Postinfectious Arthritides/  50 Reiter Syndrome/  51 Reiters Disease/  52 Reiters Syndrome/  53 Reiter Disease/  54 Arthritis, Infectious/exp  55 Infectious Arthritis/  56 Viral Arthritis/  57 Bacterial Arthritides/  58 Septic Arthritis/  59 Bacterial Arthritis/  60 Suppurative Arthritis/  61 enteropathic arthritis/  62 traumatic arthritis/  63 Arthritis, Psoriatic/exp  64 Arthritic Psoriasis/  65 Psoriatic Arthritis/  66 Psoriasis Arthropathica/  67 Psoriatic Arthropathy/  68 Psoriatic Arthropathies/  69 Arthritis/exp  70 Arthritides/  71 Polyarthritis/  72 Polyarthritides/  73 or/1-72  74 Iguratimod/  75 Alamode/  76 ('T-614' or 'C17H14N2O6S' or 'IGU').ti,ab.  77 or/74-76  78 randomized controlled trial/  79 single blind procedure/  80 crossover procedure/  81 double blind procedure/  82 or/78-81  83 73 and 77  84 82 and 83 |
