# Supplementary material for: A systematic review and meta-analysis of the efficacy and safety of iguratimod in the treatment of inflammatory arthritis and degenerative arthritis
Source: Front Pharmacol. 2024 Oct 10;15:1440584. doi: 10.3389/fphar.2024.1440584 (PMC11499590; doi:10.3389/fphar.2024.1440584)
Supplement: Supplementary file 2 [file Table2.docx]

Table S2 The details of characteristics of included studies

| **Disease** | **Study** | **Sample size** | | **Intervention** | | **Relevant outcomes** | **Mean age (years)** | | **Duration** |
| --- | --- | --- | --- | --- | --- | --- | --- | --- | --- |
|  |  | **Trial group** | **Control group** | **Trial group** | **Control group** |  | **Trial group** | **Control group** |  |
| AS | Li et al. 2021 [47] | 30 | 30 | IGU 25mg Bid + Sulfasalazine 0.5 to 1g Bid + Thalidomide 50 to 200mg Qn | Sulfasalazine 0.5 to 1g Bid + Thalidomide 50 to 200mg Qn | BASDAI | 31.24 ± 4.71 | 30.01 ± 4.68 | 24 weeks |
|  | Bai et al. 2021 [48] | 43 | 43 | IGU 25mg Bid + Sulfasalazine 1g Bid + Celecoxib 200mg Bid | Sulfasalazine 1g Bid + Celecoxib 200mg Bid | BASDAI, CRP, ESR, adverse events | 28.52±9.43 | 27.87±8.05 | 12 weeks |
|  | Lin et al. 2019 [49] | 24 | 24 | IGU 25mg Bid + Sulfasalazine 1 g Bid. + methotrexate 10 mg once a week + NSAIDs | Sulfasalazine 1 g Bid. + methotrexate 10 mg once a week + NSAIDs | BASDAI, BASFI, adverse events | 32. 71 ± 8. 80 | 28. 21 ± 6. 69 | 24 weeks |
|  | Xu et al. 2019 [50] | 21 | 21 | IGU 25mg Bid + Celecoxib 0.2 g Qd. | Sulfasalazine 1 g Bid. + Celecoxib 0.2 g Qd. | BASDAI, BASFI, ESR, CRP, adverse events | 35.1± 10.3 | 34.3± 9.5 | 24 weeks |
|  | Zeng et al 2016 [51] | 25 | 25 | IGU 25mg Bid + Meloxicam 7.5 mg Qd. | Sulfasalazine 0.75 g Tid. + Meloxicam 7.5 mg Qd. | BASDAI, CRP, adverse events | 38±12 | 40±10 | 24 weeks |
|  | Yan et al. 2021 [52] | 48 | 25 | IGU 50mg Qd + NSAIDs | NSAIDs + Placebo | BASDAI, BASFI, CRP, ESR, adverse events | 31.38 ± 7.36 | 30.28 ± 5.94 | 24 weeks |
|  | Yuan et al. 2020 [53] | 41 | 39 | IGU 25mg Bid + Etoricoxib tablets 60 mg Qd. + ibuprofen 300 mg Tid. + methotrexate 15 mg once a week | Etoricoxib tablets 60 mg Qd. + ibuprofen 300 mg Tid. + methotrexate 15 mg once a week | CRP, ESR, adverse events | 39.28 ± 5.30 | 40.08 ± 5.67 | 12 weeks |
|  | Pang et al. 2020 [54] | 39 | 39 | IGU 25mg Bid + Etanercept 25mg tiwce a week | Etanercept 25mg tiwce a week | BASDAI, ESR, CRP | 24.85±4.18 | 25.01±4.29 | 12 weeks |
|  | Zhang 2022 [55] | 35 | 34 | IGU 25mg Bid + Sulfasalazine 0.25g Bid | Sulfasalazine 0.25g Bid + Celecoxib 0.2g Qd | BASFI, ESR, CRP, adverse events | 49-75 | 48-74 | 12 weeks |
| OA | Zeng et al. 2019 [56] | 23 | 23 | IGU 25mg Bid | Glucosamine 0.5g Bid | WOMAC, VAS, IL-6, TNF-α, Adverse events | 56.4 ± 11.6 | 55.3 ± 12.7 | 12 weeks |
|  | Zhang et al. 2023 [57] | 85 | 82 | IGU 25mg Bid + Glucosamine sulfate 0.25 Tid | Glucosamine sulfate 0.25 Tid | WOMAC, VAS, IL-6, TNF-α, Adverse events | 54.82 ± 6.90 | 54.37 ± 6.48 | 8 weeks |
| RA | Han et al. 2023 [58] | 50 | 49 | IGU 25mg Bid+Leflunomide 20mg Qd | MTX 10 mg once a week+Leflunomide 20mg Qd | DAS28, CRP, ESR, RF, ACR20, ACR50, ACR70, Adverse events | 63.75-70.0 | 61.0-67.0 | 52 weeks |
|  | Wu et al. 2022 [59] | 58 | 58 | IGU 25mg Bid+MTX 10 mg once a week+ Tripterygium wilfordii polyglycosides 50 mg for the first time and 20 mg Qd after 3days | MTX 10 mg once a week+ Tripterygium wilfordii polyglycosides 50 mg for the first time and 20 mg Qd after 3days | DAS28, CRP, ESR, RF | 61.48±4.36 | 62.73±4.58 | 18 weeks |
|  | Lü et al. 2008 [60] | 185 | 95 | a: IGU 25 mg Qd; b: 25mg Bid | Placebo | ACR20, ACR50, ACR70, ESR, CRP, RF, adverse events | a: 48.05±10.30; b: 46.98±10.93 | 47.46±10.30 | 24 weeks |
|  | Hara et al. 2007 [61] | 132 | 64 | IGU 25 mg for the first 4 weeks and 50 mg for the subsequent 24 weeks | placebo | CRP, ESR, adverse events | 57.5 ± 10.8 | 57.0 ± 10.8 | 28 weeks |
|  | Mo et al. 2015 [62] | 30 | 30 | IGU 25mg Bid+MTX 15 mg once a week | MTX 15 mg once a week | ACR20, ACR50, ACR70, ESR, CRP, RF, adverse events | 31. 8 ± 8. 5 | 31. 9 ± 8. 6 | 12 weeks |
|  | Xiong et al. 2020 [63] | 51 | 51 | IGU 25mg Bid+MTX 10 mg once a week at the beginning; 12.5 mg twice a week after 2 weeks; 15 mg once a week after 4 weeks | MTX 10 mg once a week at the beginning; 12.5 mg twice a week after 2 weeks; 15 mg once a week after 4 weeks | Adverse events | 48. 21 ± 6. 04 | 48. 33 ±5. 93 | 24 weeks |
|  | Wang et al. 2022 [64] | 60 | 60 | IGU 25mg Bid+MTX 10 mg once a week | MTX 10 mg once a week | CRP, adverse events | 54 ± 14 | 55 ± 13 | 12 weeks |
|  | Dai et al. 2022 [65] | 60 | 60 | IGU 25mg Bid+MTX 7.5 mg once a week | MTX 7.5 mg once a week | DAS28, CRP, ESR, RF | 59.4±7.8 | 60.1±9.7 | 12 weeks |
|  | Deng et al. 2017 [66] | 59 | 31 | a: IGU 25mg Bid+MTX 10 mg once a week; b: IGU 25mg Bid | MTX 10 mg once a week +Leflunomide 20mg Qd | DAS28, ESR, CRP, RF, adverse events | 47.23±15.62 | | 48 weeks |
|  | Tian et al. 2020 [67] | 120 | 120 | IGU 25mg Bid+MTX 10 mg once a week | MTX 10 mg once a week +Leflunomide 20mg Qd | DAS28, ESR, CRP, RF, adverse events | 50±10 | 49±11 | 52 weeks |
|  | Fan et al. 2020 [68] | 38 | 37 | IGU 25mg Bid+MTX 10 mg once a week at the beginning; 12.5 mg once a week after 2 weeks; 15 mg once a week after 4 weeks | MTX 10 mg once a week at the beginning; 12.5 mg once a week after 2 weeks; 15 mg once a week after 4 weeks | DAS28 | 49.0± 10.1 | 48.7± 10.2 | 24 weeks |
|  | Xie et al. 2018 [69] | 39 | 39 | IGU 25mg Bid+MTX 10 mg once a week at the beginning; 12.5 mg twice a week after 2 weeks; 15 mg once a week after 4 weeks | MTX 10 mg once a week at the beginning; 12.5 mg twice a week after 2 weeks; 15 mg once a week after 4 weeks | DAS28, adverse events | 62. 89 ± 4. 57 | 62. 74 ± 3. 96 | 16 weeks |
|  | Li et al. 2019 [70] | 51 | 51 | IGU 25mg Bid+MTX 15 mg once a week | MTX 15 mg once a week | Adverse events | 74. 16 ± 2. 42 | 74. 32 ± 2. 52 | 15 weeks |
|  | Zhao et al. 2018 [71] | 36 | 36 | IGU 25mg Bid+MTX 7.5 mg once a week | MTX 7.5 mg once a week | DAS28, CRP, adverse events | 47.20±3.40 | 50.80±4.10 | 12 weeks |
|  | Ma et al. 2019 [72] | 52 | 104 | IGU 25mg Bid+Tripterygium glycosides 1.5 mg/(kg·d) | a: Prednisone + Sulfasalazine; b: Tripterygium glycosides 1.5 mg/(kg·d) | Forced vital capacity (FVC), Forced expiratory volume in 1 second (FEV1), total lung capacity (TLC), CRP, RF, adverse events | 54.7±5.1 | a: 55.6±4.9; b: 54.1±5.4 | 24 weeks |
|  | Zhao et al. 2016 [73] | 60 | 30 | a: IGU 25mg Bid+MTX 10 mg once a week; b: IGU 25mg Bid | MTX 15 mg once a week | ACR20, ACR50, ACR70, adverse events | a: 30.1 ± 2.4; b: 29.3 ± 2.7 | 28.1 ± 3.4 | 24 weeks |
|  | Meng et al. 2017 [74] | 60 | 60 | IGU 25mg Bid+MTX 10 mg once a week | MTX 10 mg once a week | RF, CRP, adverse events | 64.83 ±9.41 | 64.31 ±8.22 | 12 weeks |
|  | Xia et al. 2016 [75-76] | 100 | 50 | a: IGU 25mg Bid+MTX 10 mg once a week; b: IGU 25mg Bid | MTX 10 mg once a week | ESR, CRP | 46.63±10.61 | | 24 weeks |
|  | Qi et al. 2019 [77] | 40 | 40 | IGU 25mg Bid+MTX 7.5 mg once a week at the beginning, gradually increase to 10mg within 4 weeks | MTX 7.5 mg once a week at the beginning,Gradually increase to 10mg within 4 weeks | ACR20, ACR50, ACR70, ESR, CRP, adverse events | 25-65 |  | 24 weeks |
|  | Zhao et al. 2017 [78] | 63 | 33 | a: IGU 25mg Bid; b: IGU 25mg Bid+MTX 10 mg once a week | MTX 10 mg once a week | ACR20, ACR50, ACR70, DAS28, ESR, CRP, RF, adverse events | a: 46.46 ± 11.01; b: 45.97 ± 10.75 | 46.31 ± 10.89 | 24 weeks |
|  | Hu et al. 2014 [79] | 20 | 20 | IGU 25mg Bid | MTX 10 mg once a week | DAS28, ACR20, adverse events | 47.3±13.5 | 46.2±15.8 | 24 weeks |
|  | Chen et al. 2018 [80] | 60 | 60 | IGU 25mg Bid+MTX 10 mg once a week | MTX 10 mg once a week | CRP, adverse events | 45.7±5.4 | 45.9±4.8 | 24 weeks |
|  | Xia et al. 2020 [81] | 50 | 50 | IGU 25mg Bid+MTX 7.5 mg once a week at the beginning, increase by 2.5mg per week, with a final dose of 15mg | MTX 7.5 mg once a week at the beginning, increase by 2.5mg per week, with a final dose of 15mg+Tripterygium glycosides 1-1.5mg/kg | ESR, CRP | 53.73±2.78 | 53.62±2.45 | 12 weeks |
|  | Tian et al. 2017 [82] | 58 | 58 | IGU 25mg Bid+MTX 10 mg once or twice a week | MTX 10 mg once or twice a week | DAS28, ESR, CRP, adverse events | 52.6±7.6 | 49.7±8.4 | 24 weeks |
|  | Xu et al. 2017 [83] | 42 | 41 | IGU 25mg Bid+MTX 7.5-20mg once a week | MTX 7.5-20mg once a week | DAS28, ESR, CRP | 46.34± 2.29 | 46.19± 2.57 | 48 weeks |
|  | Shi et al. 2015 [84] | 30 | 30 | IGU 25mg Bid+MTX 10 mg once a week at the beginning;12.5 mg twice a week after 4 weeks | MTX 10 mg once a week at the beginning;12.5 mg twice a week after 4 weeks | DAS28, ESR, CRP, ACR20, ACR50, ACR70, adverse events | 48.9 ± 12.2 | 48.4 ± 10.2 | 24 weeks |
|  | Wang et al. 2019 [85] | 47 | 46 | IGU 25mg Bid+MTX 15 mg once a week | MTX 15 mg once a week | CRP, RF, ESR, DAS28 | 48. 13 ± 6. 40 | 47. 83 ± 6. 37 | 24 weeks |
|  | Hara et al. 2014 [86-87] | 164 | 68 | IGU 25mg Qd for the first 4 weeks of the extension period 25 mg Bid for the subsequent 20 week+MTX 6-8 mg once a week | MTX 6-8 mg once a week+placebo | ACR20, ACR50, ACR70, CRP, RF, DAS28, adverse events | 54.8±9.9 | 53.5±10.0 | 24 weeks |
|  | Rao et al. 2014 [88] | 60 | 30 | a: IGU 25mg Bid; b: IGU 25mg Qd | MTX 10 mg once a week | ACR20, ACR50, ACR70 | 42.6±5.2 |  | 12 weeks |
|  | Xu et al. 2015 [89] | 72 | 38 | a: IGU 25mg Bid+MTX 7.5-20mg once a week; b: IGU 25mg Bid | MTX 7.5-20mg once a week | ESR, CRP, RF, adverse events | a: 46.10±17.09; b: 44.71±9.32 | 43.28±10.46 | 48 weeks |
|  | Bi et al. 2019 [90] | 30 | 30 | IGU 25mg Bid+MTX 10 mg once a week | MTX 10 mg once a week +Leflunomide 20mg Qd | DAS28, adverse events | 53.10±12.90 | 54.60±11.88 | 12 weeks |
|  | Sun and Li 2022 [91] | 43 | 43 | IGU 25mg Bid+MTX 10 mg once a week | MTX 10 mg once a week | Adverse events | 49.05 ± 4.32 | 48.96 ± 5.24 | 24 weeks |
|  | Yan et al. 2018 [92] | 35 | 35 | IGU 25mg Bid+MTX 10 mg once a week | MTX 10 mg once a week | Adverse events | 56 ± 7 | 56 ± 7 | 24 weeks |
|  | Meng et al. 2016 [93] | 30 | 30 | IGU 25mg Bid+MTX 15 mg once a week | MTX 15 mg once a week | DAS28, adverse events | 41.6±20.3 | 45.1±19.2 | 16 weeks |
|  | Duan et al. 2015 [94] | 30 | 30 | IGU 25mg Bid+MTX 10 mg once a week at the beginning, gradually increase to 12.5mg within 4 weeks | MTX 10 mg once a week at the beginning, gradually increase to 12.5mg within 4 weeks | ESR, CRP, DAS28, adverse events | 48.9±12.2 | 48.4±10.2 | 24 weeks |
|  | Xiong et al. 2015 [95] | 30 | 28 | IGU 25mg Bid+MTX 10 mg once a week | MTX 10 mg once a week | RF, CRP, ESR, DAS28, adverse events | 56±12 | 51±13 | 24 weeks |
|  | Lu et al. 2009 [96] | 326 | 163 | a: IGU 25 mg for the first 4 weeks and 50 mg for the subsequent 20 weeks; b: IGU 25mg Bid | MTX 10 mg/week for the first 4 weeks and 15 mg/week for the subsequent 20 weeks | ACR20, ACR50, ACR70, ESR, CRP, RF, adverse events | a: 46.0 ± 10.6; b: 45.9 ± 10.4 | 47.2 ± 11.0 | 24 weeks |
|  | Ju et al. 2020 [97] | 58 | 58 | IGU 25mg Bid+MTX 10 mg once a week | MTX 10 mg once a week | DAS28, ESR, CRP, RF | 42.31±13.78 | 41.87±13.94 | 24 weeks |
|  | Meng et al. 2015 [98] | 33 | 33 | IGU 25mg Bid+MTX 10 mg once a week | MTX 10 mg once a week+Leflunomide 10mg Qd | DAS28, ACR20, ACR50, ACR70, adverse events | 44. 2 ± 20. 5 | 41. 7 ± 22. 8 | 16 weeks |
|  | Li et al. 2016 [99] | 44 | 40 | IGU 25mg Qd+MTX 7.5-10 mg once a week | MTX 7.5-10 mg once a week+Tripterygium glycosides 20mg Bid | DAS28, ESR, CRP, adverse events | 60-77 | 60-82 | 12 weeks |
|  | Li et al. 2020 [100] | 20 | 13 | IGU 25mg Bid+MTX 10 mg once a week | MTX 10 mg once a week+Adalimumab 40mg once every 2 weeks | DAS28 | 58±11 | 55±11 | 24 weeks |
|  | Mo et al. 2018 [101] | 30 | 30 | IGU 25mg Bid+MTX 10 mg once a week | MTX 10 mg once a week+Tripterygium glycosides 20mg Bid | DAS28, ESR, CRP, CCP, RF, adverse events | 45 ± 11.6 | 43.3 ± 10.25 | 12 weeks |
|  | Shi et al. 2023 [102] | 62 | 62 | IGU 25mg Bid+MTX 10 mg once a week at the beginning; 12.5 mg once a week after 2 weeks+Hydroxychloroquine sulfate 200mg | MTX 10 mg once a week at the beginning; 12.5 mg once a week after 2 weeks+Hydroxychloroquine sulfate 200mg | DAS28, ESR, RF, Adverse events | 48. 56±10. 34 | 49. 86±12. 35 | 24 weeks |
